# Exploring Phytochemical Mechanisms in the Prevention of Cholesterol Dysregulation: A Review

**DOI:** 10.1021/acs.jafc.3c09924

**Published:** 2024-03-22

**Authors:** Monthana Weerawatanakorn, Sudthida Kamchonemenukool, Yen-Chun Koh, Min-Hsiung Pan

**Affiliations:** † Department of Agro-Industry, Naresuan University, 99 Moo 9, Thapho, Muang, Phitsanulok 65000, Thailand; ‡ Centre of Excellence in Fats and Oils, Naresuan University Science Park, 99 M 9, Thapho, Muang, Phitsanulok 65000, Thailand; § Institute of Food Science and Technology, National Taiwan University, Taipei 10617, Taiwan; ∥ Department of Medical Research, China Medical University Hospital, China Medical University, Taichung City 40447, Taiwan; ⊥ Department of Health and Nutrition Biotechnology, Asia University, Taichung City 41354, Taiwan

**Keywords:** cholesterol, cholesterol dysregulation, phytochemical, bile acid, gut microbiota

## Abstract

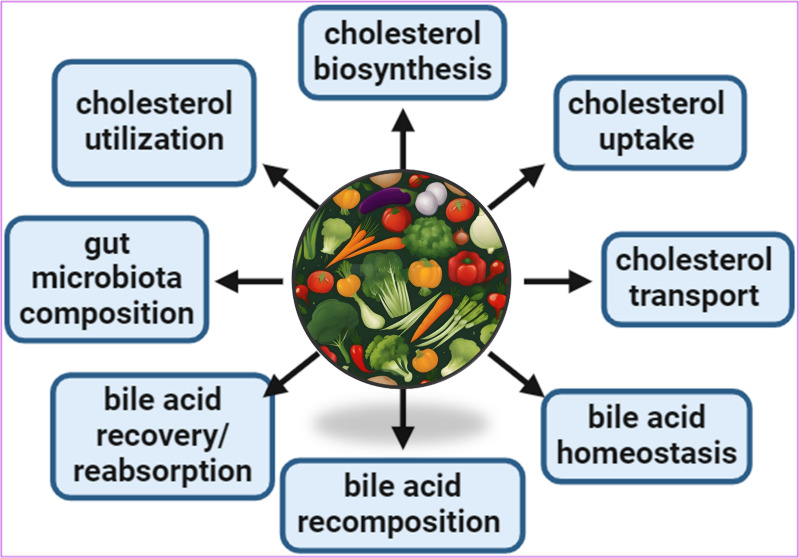

Although cholesterol plays a key role in many physiological processes, its dysregulation can lead to several metabolic diseases. Statins are a group of drugs widely used to lower cholesterol levels and cardiovascular risk but may lead to several side effects in some patients. Therefore, the development of a plant-based therapeutic adjuvant with cholesterol-lowering activity is desirable. The maintenance of cholesterol homeostasis encompasses multiple steps, including biosynthesis and metabolism, uptake and transport, and bile acid metabolism; issues arising in any of these processes could contribute to the etiology of cholesterol-related diseases. An increasing body of evidence strongly indicates the benefits of phytochemicals for cholesterol regulation; traditional Chinese medicines prove beneficial in some disease models, although more scientific investigations are needed to confirm their effectiveness. One of the main functions of cholesterol is bile acid biosynthesis, where most bile acids are recycled back to the liver. The composition of bile acid is partly modulated by gut microbes and could be harmful to the liver. In this regard, the reshaping effect of phytochemicals on gut microbiota has been widely reported in the literature for its significance. Therefore, we reviewed studies conducted over the past 5 years elucidating the regulatory effects of phytochemicals or herbal medicines on cholesterol metabolism. In addition, their effects on the recomposition of gut microbiota and bile acid metabolism due to modulation are discussed. This review aims to provide novel insights into the treatment of cholesterol dysregulation and the anticipated development of natural-based compounds in the near and far future.

## 1 Introduction

Many organizations have provided definitions of metabolic syndrome (MetS), the meaning of which has been progressively developed to be as comprehensive as possible—in chronological order, these are the World Health Organization (WHO), in 1998; the European Group for the Study of Insulin Resistance (EGIR), in 1999; the National Cholesterol Education Program Adult Treatment Panel III (NCEP ATP III), in 2001; and the International Diabetes Federation (IDF), in 2005.^1^ Among them, the NCEP ATP III definition provides one of the most widely used sets of criteria for MetS since it includes the crucial features of hyperglycemia/insulin resistance, visceral obesity, atherogenic dyslipidemia, and hypertension.^2^ According to this panel, in MetS, three or more of the following five criteria must be met: waist circumference over 40 in. (men) or 35 in. (women), blood pressure over 130/85 mmHg, fasting triglyceride (TG) level over 150 mg/dL, fasting high-density lipoprotein (HDL) cholesterol level less than 40 mg/dL (men) or 50 mg/dL (women), and fasting blood sugar level over 100 mg/dL.^2^

Most of the above-mentioned criteria are correlated with the incidence of hyperlipidemia, a health condition characterized by the presence of excess plasma lipids (cholesterol, triglycerides, phospholipids, etc.) and lipoproteins (high-, low-, and very-low-density lipoproteins).^3^ Hyperlipidemia is subclassified into hypercholesterolemia and hypertriglyceridemia, where an increase in plasma triglyceride levels is notably related to hypercholesterolemia. Although cholesterol is an essential precursor for biological molecules, including steroid hormones, bile acids, oxysterols, and vitamin D, the accumulation of free cholesterol has adverse effects on metabolism and health.^4^ A low-saturated-fat diet is always advised as a strategic approach to lower plasma cholesterol.^5^ However, due to the inadequate cholesterol-lowering effects achieved from following dietary recommendations alone, statins (including pravastatin, simvastatin, atorvastatin, and rosuvastatin) have been employed and are efficacious in reducing plasma cholesterol, especially low-density lipoprotein (LDL) cholesterol.^5^ Nevertheless, statin drugs may lead to adverse effects in patients using multiple medications, including muscle damage, renal failure, and myopathy; in addition, scholars have suggested some risk factors associated with statin toxicity, such as alcohol abuse, frailty, multisystem diseases, gender, and age.^6^ Many preclinical and clinical studies have reported that nutraceuticals and phytochemicals from plant-based products exhibit cholesterol-lowering properties and can be potentially used as alternative medication for lowering the risks of atherosclerosis-related diseases.^7,8^ Therefore, researchers have encouraged the development of plant-based health products, including health food, functional food, and dietary supplements, containing nutraceuticals with hypocholesterolemic activity (as proven by their pharmacological effects) as a sustainable intervention to retard cholesterol increase.^9^ This review focuses on recent strategies and approaches to manage hypercholesterolemia using phytochemicals within 2019–2023.

## 2 Regulation of Cholesterol Homeostasis

In a recent review, Duan et al. (2022) summarized the key steps and molecular mechanisms involved in the regulation of cholesterol homeostasis—including cholesterol biosynthesis, uptake, transport, utilization, and excretion—and the effect of epigenetic modulation on cholesterol metabolism.^10^ The subsequent sections focus on phytochemicals or plant-based products exhibiting regulatory effects on the molecular mechanisms of cholesterol homeostasis; most of the studies discussed in this review have been published between 2020 and 2023.

### 2.1 Effect of Phytochemicals on Cholesterol Biosynthesis

**Figure 1:**
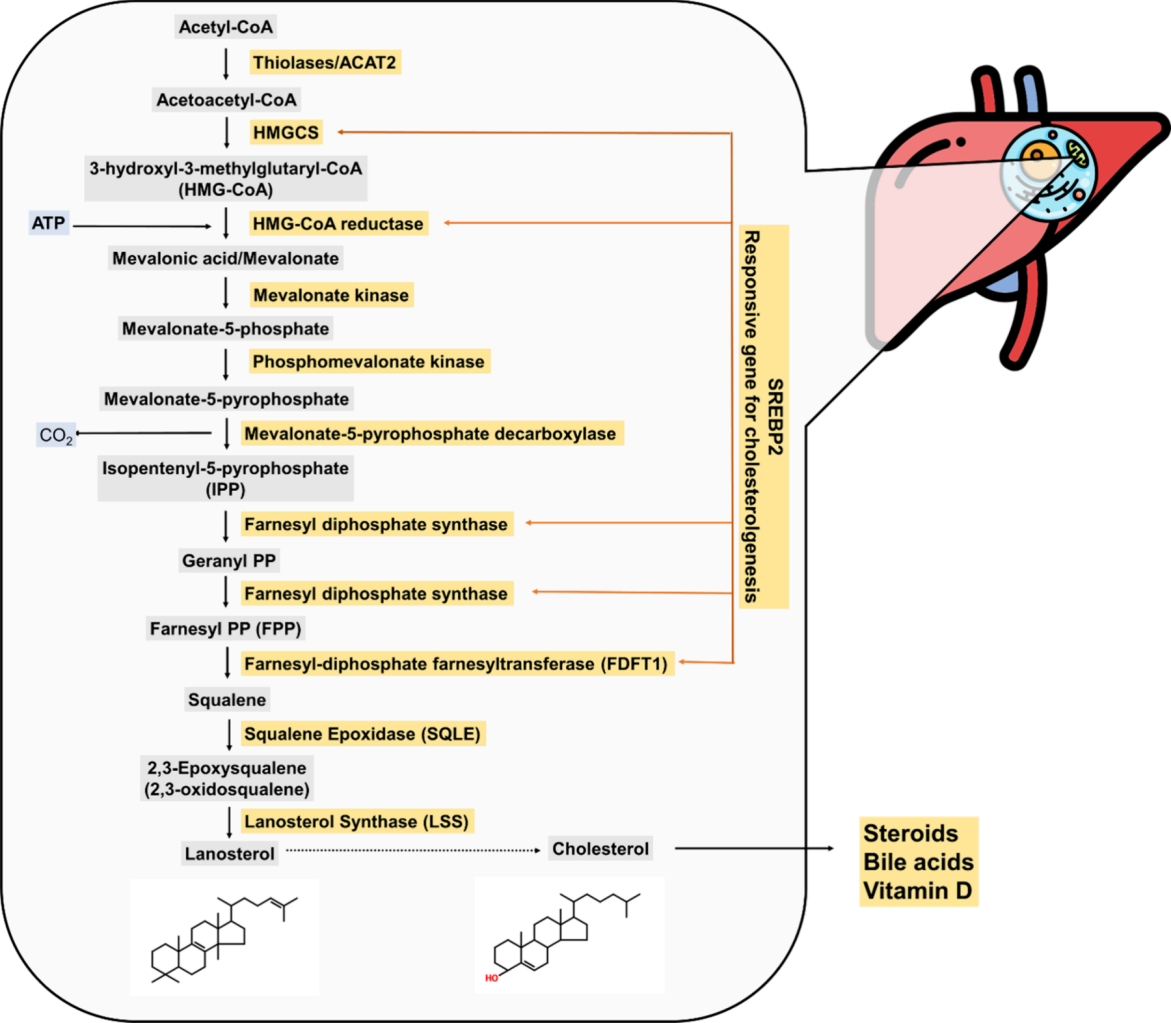
Process of hepatic cholesterol biosynthesis, adapted from Sitaula and Burris (2016).^71^ Copyright 2016 Academic Press.

The inhibition of cholesterol synthesis by phytoestrogens and phytochemicals has been suggested as a strategy to reduce blood cholesterol.^11^ This section discusses the regulatory effect of these natural compounds on the enzymes involved in controlling cholesterol biosynthesis, including sterol regulatory element-binding proteins (SREBPs), 3-hydroxy-3-methylglutaryl-CoA synthase 1 (HMGCS1), 3-hydroxy-3-methylglutaryl-coenzyme A reductase (HMGCR), and farnesyl-diphosphate farnesyltransferase 1 (FDFT1; Figure 1 and Table 1).

**Table 1:**
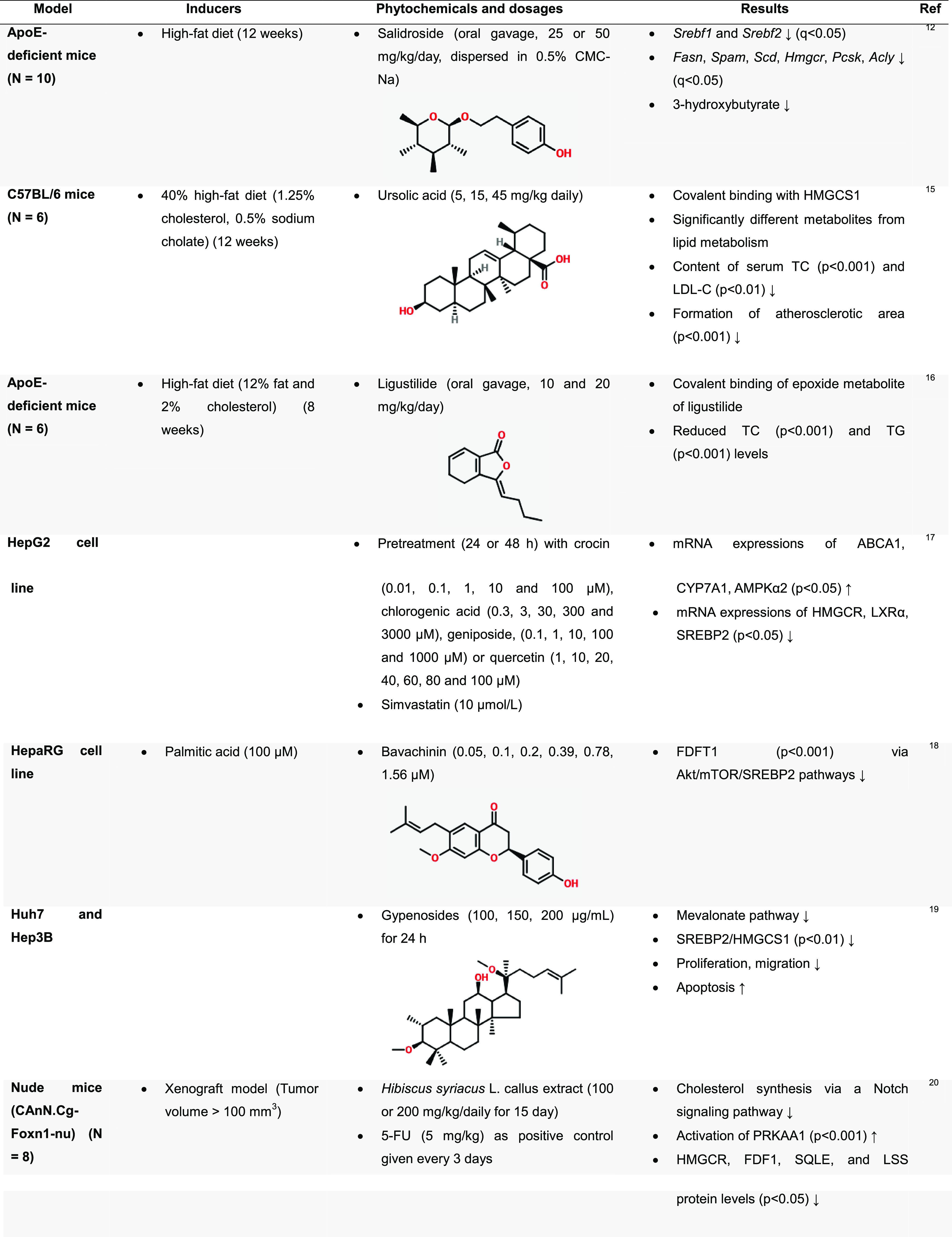

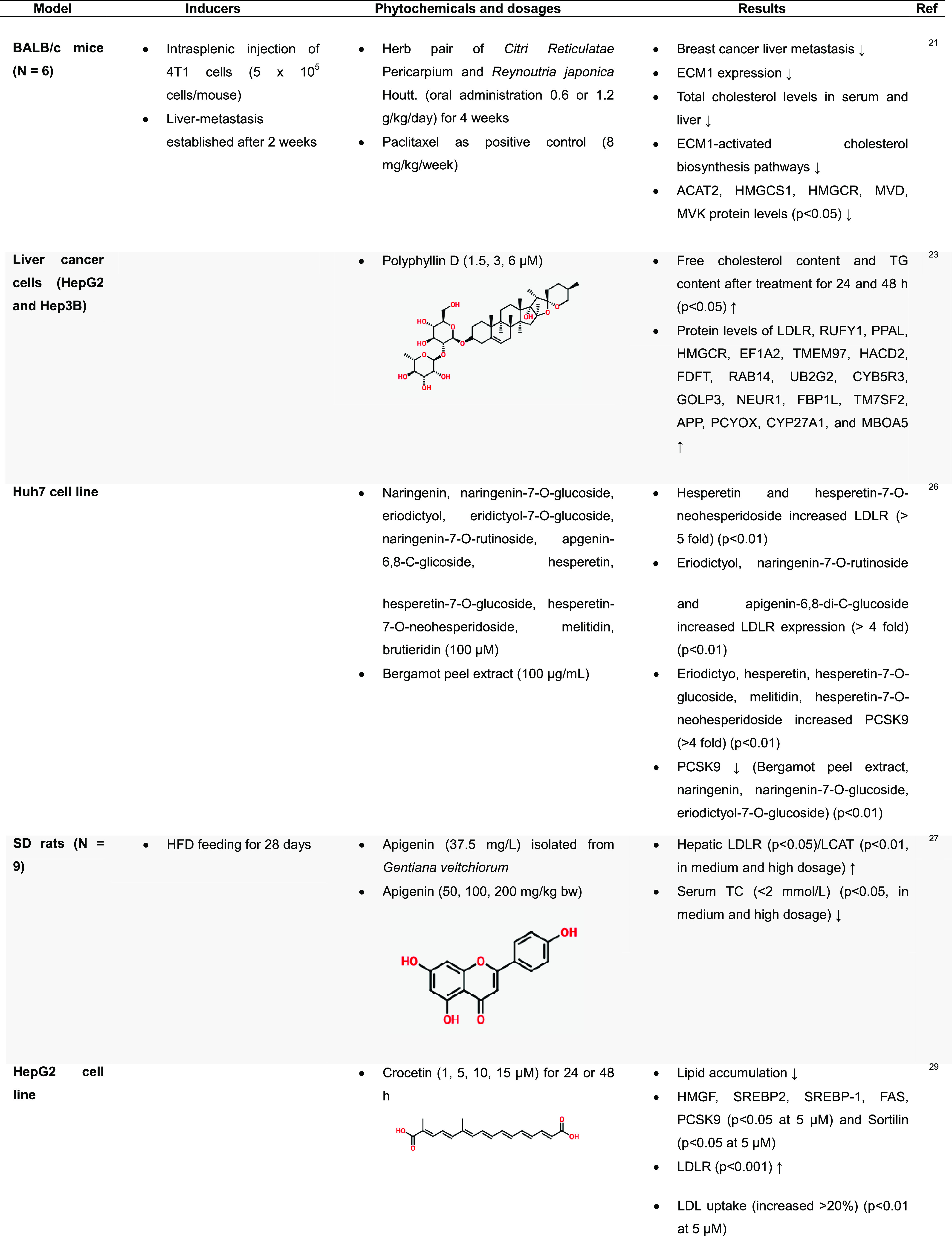

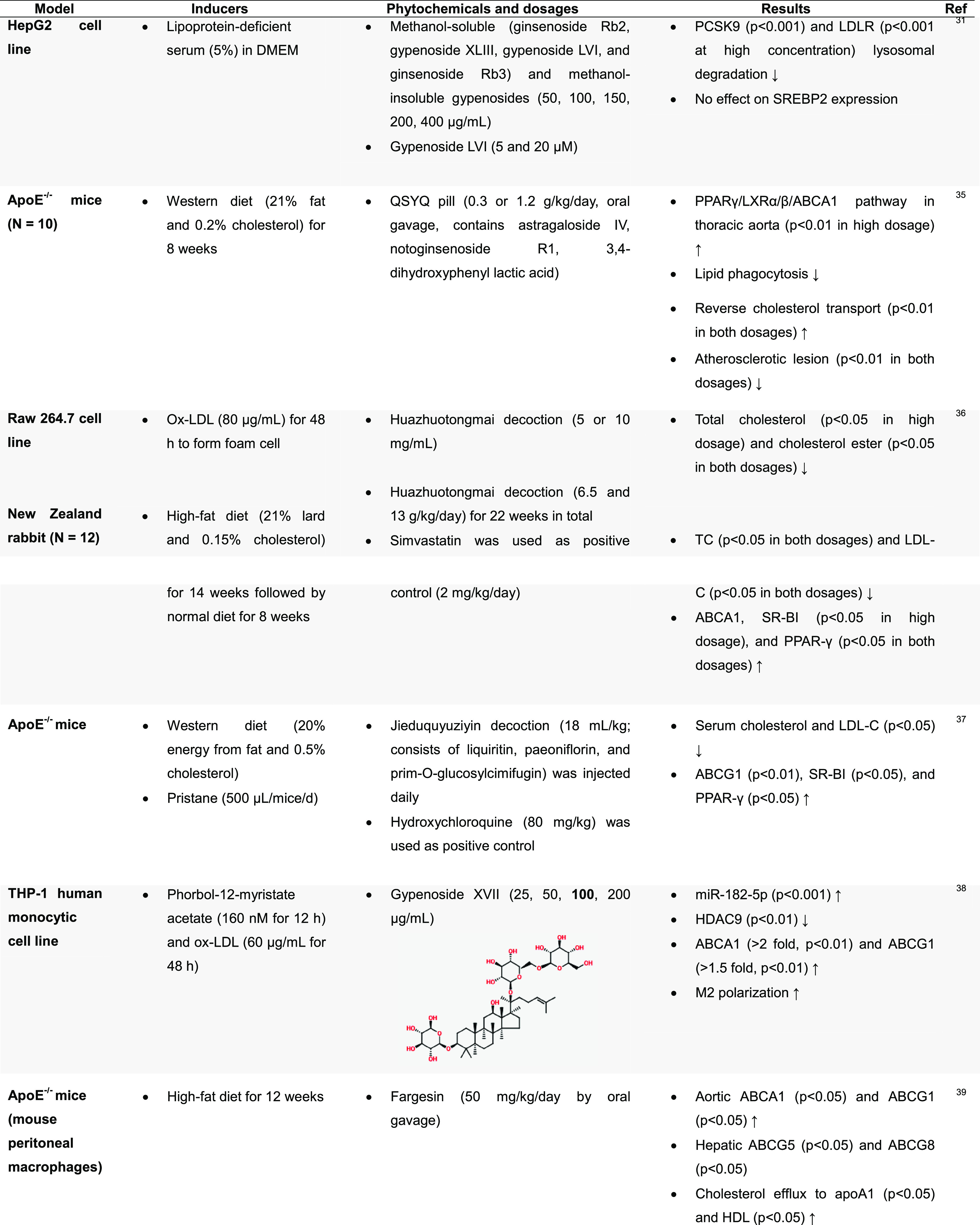

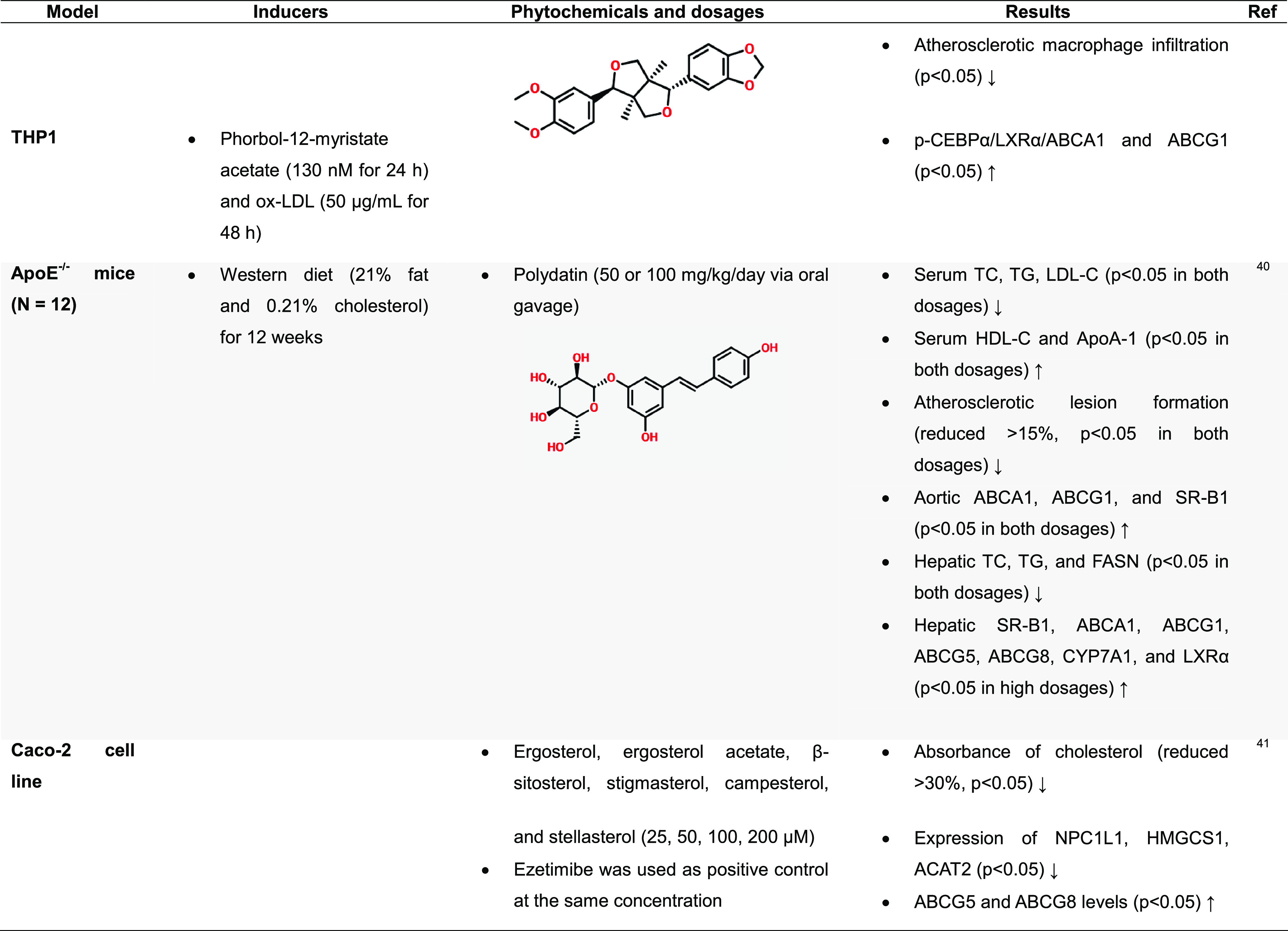
Effect of Phytochemicals on Cholesterol Metabolism in Different Disease Models

#### 2.1.1 Sterol Regulatory Element-Binding Proteins (SREBPs)

SREBPs regulate several downstream genes, including those for fatty acid synthase (*Fasn*), glycerol-3-phosphate acyltransferase (*Gpam*), stearoyl-CoA desaturase (*Scd*), and proprotein convertase subtilisin/kexin type 9 (*Pcsk9*).^12^ The inhibition of these target genes via *Srebf1* and *Srebf2* downregulation by intervening salidroside, a type of phenylethanoid glycoside found in *Rhodiola* and *Ligustrum* plant species, has been demonstrated by Song et al. (2021). Salidroside also inhibits the acetyl-CoA-producing enzyme encoded by the ATP citrate lyase gene (*Acly*) and leads to an increase in the production of 3-hydroxybutyrate (a ketone body) due to fatty acid degradation in apoE-deficient mice, resulting in enhanced glycerolipid and glycerophospholipid metabolism.^12^ Fuzhuan brick tea, a postfermented tea, has been produced in China since 1860 and involves fungal fermentation, which could lead to an increment in organic acids and tea pigments; its inhibitory effects on obesity in different models have been previously reported.^13^ Furthermore, Fuzhuan brick tea has recently been found to reduce fat storage by suppressing the SREBP/MDT-15/FAT-2 pathway under normal cholesterol intake but may promote fat storage via the same pathway under a high-cholesterol dietary intake in the *C. elegans* model.^13^ Since SREBPs are important in cholesterol synthesis and metabolism, they are considered a major target for suppressing cholesterol biosynthesis.

#### 2.1.2 3-Hydroxy-3-methylglutaryl-CoA Synthase 1 (HMGCS1) and 3-Hydroxy-3-methylglutaryl-coenzyme A Reductase (HMGCR)

HMGCS1 is a key enzyme in free cholesterol synthesis and is regulated by sterol regulatory element-binding protein 2 (SREBP2) and signal transducer and activator of transcription 1 (STAT1).^14^ The inhibition of HMGCS1 by direct binding with phytochemicals could thus be considered a new strategy in cholesterol reduction. Ursolic acid, a phytochemical widely found in medicinal herbs such as rosemary and thyme, has been previously shown to alleviate hypercholesterolemia and cardiovascular disease.^15^ Ma et al. (2022) revealed that ursolic acid could covalently bind to the thiol of Cys129 in HMGCS1, resulting in the inhibition of its catalytic activity and reduced precursor generation in cholesterol biosynthesis.^15^ Similarly, ligustilide, a natural phthalide derivative found in *Ligusticum striatum* and *Angelica sinensis*, also exhibits a regulatory effect on lipid metabolism by irreversibly binding to Cys129 of HMGCS1 after its metabolization to the intermediate 6,7-epoxyligustilide.^16^

HMGCR is the main target of statins, a class of cholesterol-lowering drugs. HMGCS converts acetoacetyl-CoA into 3-hydroxy-3-methylglutaryl-CoA (HMG-CoA), which is subsequently converted into mevalonate by HMGCR. Research has demonstrated that crocin, chlorogenic acid, geniposide, and quercetin intervention could reduce lipid deposition in a cell model by promoting the expression of cholesterol-metabolism-related genes and the suppression of SREBP2; furthermore, the combination of these four compounds exhibits a synergistic effect in inhibiting HMGCR expression.^17^ In addition, the drug simvastatin, used as a positive control, could not induce ATP-binding cassette (ABC) A1 and AMP-activated protein kinase (AMPK) α mRNA levels, as the natural compounds did. Unlike already-developed drugs, phytochemicals may exert their regulatory effects on multiple targets; therefore, further studies are needed to confirm the effect of phytochemicals as potential drugs for cholesterol dysregulation.

#### 2.1.3 Farnesyl-Diphosphate Farnesyltransferase 1 (FDFT1, Squalene Synthase)

In addition to HMGCS1, other enzymes involved in cholesterol synthesis and regulated by SREBPs could be inhibited by phytochemicals. Research has shown that bavachinin, a natural compound isolated from the traditional Chinese medicine *Fructus Psoraleae*, exhibits lipid-lowering effects by acting as a peroxisome proliferator-activated receptor (PPAR) agonist and inhibits squalene synthase/FDFT1 via the suppression of the protein kinase B (Akt)/mammalian target of rapamycin (mTOR)/SREBP2 signaling pathway.^18^

### 2.2 Phytochemical Inhibition of Cancer-Related Effects of Cholesterol Biosynthesis

In addition to inhibiting metabolic diseases, blocking cholesterol biosynthesis could serve as a potential cancer prevention strategy. Gypenosides, the major constituent of *Gynostemma pentaphyllum*, disrupt cholesterol biosynthesis via HMGCS1 inhibition, consequently suppressing the proliferation and migration of the Huh-7 and Hep3B cell lines.^19^ The suppressive effect of phytochemicals on cholesterol biosynthesis has been suggested in a colorectal cancer model. The callus extract of *Hibiscus syriacus* L., which is rich in fumaric acid and denatonium benzoate, was found to mediate Notch signaling, which subsequently suppressed cholesterol synthesis.^20^ Moreover, the proteins related to cholesterol biosynthesis, including HMGCR, FDFT1, squalene epoxidase (SQLE), and lanosterol synthase (LSS), were downregulated due to a reduction in Notch signaling and the activation of AMPK signaling. Notably, the callus extracts with the candidate compounds, such as fumaric acid, succinic acid, and denatonium benzoate, showed no toxicity toward normal cells; therefore, they could serve as potential candidates for cholesterol biosynthesis inhibition.

Wang et al. (2023) demonstrated that the extracellular matrix protein 1 (ECM1), elevated levels of which have been identified in malignant epithelial tumors, could regulate cholesterol biosynthesis and consequently trigger angiogenesis and cancer malignancy.^21^ Notably, the herb pair of *Citri Reticulatae* Pericarpium and *Reynoutria japonica* Houtt. have exhibited an inhibitory effect on target genes involved in the cholesterol metabolic process, including HMGCS1, FDFT1, mevalonate diphosphate decarboxylase (MVD), SQLE, and HMGCR, via ECM1 suppression. Studies have also shown that KRAS mutations in lung adenocarcinomas stop cholesterol efflux, thereby promoting tumor growth. Therefore, cholesterol removal via the promotion of the cholesterol efflux pathway could present a potential strategy to limit tumor growth.^22^

### 2.3 Promotion of Cholesterol Biosynthesis by Phytochemicals

Some phytochemicals have been shown to promote cholesterol biosynthesis. For example, Chen et al. (2023) demonstrated that polyphyllin D, a steroidal saponin found in the rhizomes of *Paris polyphylla*, could induce cholesterol biosynthesis in liver cancer cells, with upregulation of the proteins involved, including the low-density lipoprotein receptor (LDLR, 22.17-fold change), RUN and FYVE domain-containing protein 1 (RUFY1, 5.92-fold change), PPAL and HMGCR (an approximately 4-fold change), etc.^23^ Although polyphyllin D has shown a disruptive effect on cholesterol biosynthesis in liver cancer cells, the regulatory effect on the cholesterol biosynthesis of normal hepatic cells should be addressed in future studies.

### 2.4 Effect of Phytochemicals on Cholesterol Uptake and Transport

**Figure 2:**
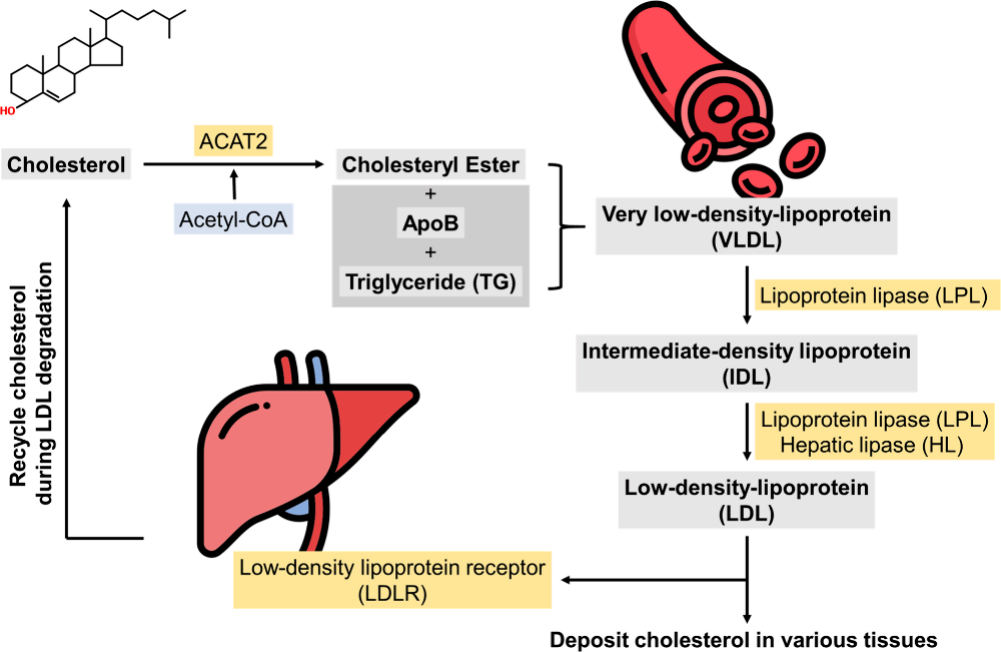
Cholesterol recycling and transport by LDL, adapted from Latimer et al. (2016).^72^ Copyright 2016 Springer.

As commonly known, low-density lipoprotein-cholesterol (LDL-C) is compulsory for cholesterol transport to various organs, though the LDL**-**C level presents a causal risk factor for atherosclerosis.^24^ Regulating cholesterol uptake and excretion to maintain host cholesterol homeostasis could represent a strategy for the pharmacological treatment of related diseases. This section discusses the phytochemicals that could potentially regulate cholesterol uptake (Figure 2) and transport.

#### 2.4.1 Cholesterol Uptake

LDLR is negatively regulated by PCSK9, and its reduction in the liver could lead to increased plasma LDL cholesterol, leading, in turn, to hypercholesterolemia.^25^ Therefore, the regulation of these proteins by inducing LDLR expression with PCSK9 inhibition could potentially control hepatic cholesterol uptake.

The methanol extract (50% methanol) of the bergamot fruit contains several glycosidic and nonglycosidic flavonoids, with naringenin-7-*O*-rutinoside and apigenin-6,8-C-glucoside exhibiting a greater effect on inducing LDLR expression among the compared compounds.^26^ Moreover, bergamot peel extract can reduce PCSK9 and its transcription factor, hepatocyte nuclear factor 1 (HNF1-α), resulting in LDL uptake in Huh7 cell lines. Other flavonoids, such as a major compound isolated from the *Gentiana veitchiorum* flower, have exhibited an ameliorative effect on the liver by promoting the LDLR-lecithin-cholesterol acyltransferase (LCAT) signaling pathway and, thereby, preventing hepatic oxidative damage.^27^ Notably, PCSK9 is the transcriptional target of SREBP2 and subsequently reduces LDLR levels; AMPK activation could be responsible for the reduction of SREBP2/PCSK9, thus preserving LDLR expression.^28^ In another study, as a key receptor able to facilitate PCSK9 transport, sortilin was inhibited by crocetin and exerted a hypocholesterolemic effect via the preservation of LDLR expression in the HepG2 cell line.^29^

As previously mentioned, SREBP2 inhibition is a potential strategy to suppress cholesterol biosynthesis. However, some studies have revealed that its activation could be beneficial. For instance, drugs such as simvastatin can promote the levels of SREBP2 and LDLR to mitigate hyperlipidemia.^30^ Both PCSK9 and LDLR are transcriptionally regulated by SREBP2 to control lipid metabolism and maintain lipid homeostasis; therefore, the regulatory effect of phytochemicals on SREBP2, which may consequently affect PCSK9 and LDLR levels, should be evaluated. A study in 2021 demonstrated the regulatory effect of gypenosides on hepatic LDL, where no significant effect was observed in the expression of SREBP2, while the LDLR expression was upregulated, and the PCSK9 level was suppressed.^31^ The authors suggested that the regulation of PCSK9 and LDLR could be stimulated by SREBP-independent pathways.

Notably, scholars have indicated that berberine, a cholesterol-lowering drug, can selectively facilitate the growth of *Blautia producta* in mice guts, where the enrichment could lead to an LDL-lowering effect via LDLR promotion.^32^ The effect was further proven by treating the HepG2 cell line with *Blautia producta*, resulting in a LDL absorption. These findings indicate the multiple effects of phytochemicals on cholesterol uptake, though further evidence is required in this regard.

#### 2.4.2 Cholesterol Transport

Reverse cholesterol transport (RCT) involves the collection of excess cholesterol by HDL and its delivery to the liver, where it is broken down and excreted.^33^ ABC transporter proteins from the A subfamily are responsible for mediating RCT; on the other hand, those from the G subfamily are responsible for cholesterol efflux, with the most well-known member being the ABCG5-ABCG8 heterodimer.^34^

Some phytochemicals from Chinese traditional herbs employ this mechanism. For instance, the Qishen Yiqi Chinese herb pill, consisting of astragaloside IV, salvianolic acid B, and notoginsenoside R1, increases ABCA1 expression via the PPAR-γ/liver X receptor (LXRα/β) pathway to promote the RCT signaling pathway, leading to the attenuation of atherosclerotic lesions.^35^ In 2023, researchers reported that the Huazhuotongmai decoction (traditional Chinese medicine), containing up to 30 different bioactive compounds, promoted RCT by upregulating the expression of ABCA1, scavenger receptor class B type I (SR-BI), and PPAR-γ in rabbits fed a high-fat diet, resulting in an antiatherosclerotic effect.^36^ In addition, the Jieduquyuziyin prescription (Chinese medicine) promotes cholesterol efflux via a similar pathway in mice livers.^37^ Based on the above-mentioned studies, RCT has recently been identified as a potential mechanism of defense against atherosclerosis progression.

Gypenoside XVII, a natural compound isolated from the traditional herbal medicine *Gynostemma pentaphyllum* Makino, has been shown to promote the expression of ABCA1 and ABCG1 via the inhibition of histone deacetylase 9 (HDAC9) expression by miR-182-5p in lipid-loaded macrophages.^38^ In addition, activation of miR-182-5p/HDAC9 by gypenoside XVII to promote cholesterol efflux potentially prevented oxidized low-density lipoprotein (ox-LDL)-induced macrophages and avoided atherosclerotic plaque formation. Similarly, fargesin, a neolignan isolated from Magnolia plants, exhibited a promoting effect on RCT in both apoE^–/–^ mice and ox-LDL-induced macrophages.^39^ Notably, fargesin upregulated ABCA1 and ABCG1 via the CCAAT/enhancer binding protein (CEBP)α/LXRα pathway and specifically increased the level of phosphorylation of CEBPα in Ser21 in THP1-derived macrophages.

Polydatin, also known as 3,4′,5-trihydroxystilbene-3-β-mono-d-glucoside, is a bioactive compound detected in various plants, such as grapes and peanuts. By inducing cholesterol efflux via ABCA1, ABCG1, and SR-BI in aortic macrophages, polydatin reduced the formation of foam cells in the aorta.^40^ In addition, it promoted the expression of ABCG5/G8 and CYP7A1 for the secretion of cholesterol. Yuan et al. (2020) showed that phytosterols (ergosterol, stigmasterol, β-sitosterol, campesterol, ergosterol acetate, and stellasterol) regulate the uptake and efflux of cholesterol. Specifically, NPC1-like intracellular cholesterol transporter 1 (NPC1L1) was inhibited to reduce the absorption of cholesterol, while ABCG5/G8 levels were enhanced to increase its excretion.^41^ Although the cholesterol-lowering effect via multiple mechanisms of phytosterols has been previously reviewed,^42^ the authors of this study suggested that the types and positions of functional groups on the phytosterols might contribute to their cholesterol-lowering effects. On the other hand, a recent study has revealed that phytosterol ferulate, a compound naturally present in certain grains (albeit in low amounts), demonstrated a lipid-lowering effect that proved to be more effective than merely combining phytosterols and ferulic acid.^43^ Lower levels of serum and hepatic TC were observed in obese mice that were supplemented with the sample; however, the underlying mechanism still needs further clarification. Moreover, several structural modified plant sterols have been identified which have high potential to be developed into cholesterol controlling supplements.^44^

Certain other members of the ABC transporter family play an important role in cholesterol transport in various tissues; the regulation of these transporters and their roles in disease amelioration should be investigated in future studies.

## 3 Association of Bile Acids with Cholesterol Homeostasis

**Figure 3:**
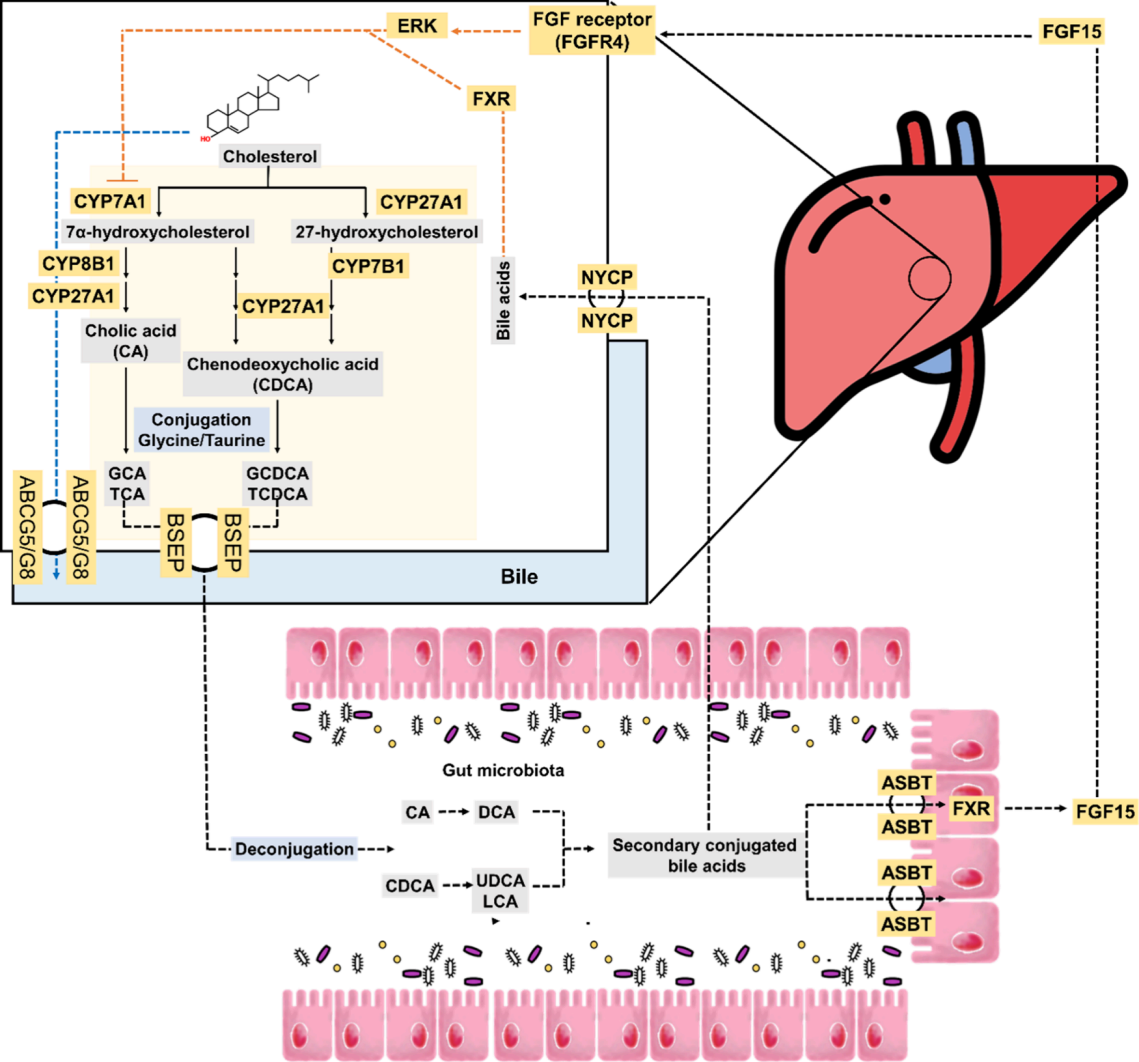
Hepatic bile acid metabolism and reabsorption, adapted from Salic et al. (2019).^73^ Copyright 2019 PLoS.

Synthesized in the liver from cholesterol, bile acids are conserved molecules and are essential for lipid homeostasis. Bile acid synthesis is a multistep process involving up to 17 enzymes located in different cellular organelles. The liver is responsible for the catabolism of cholesterol into bile acids since it contains all the required enzymes for the process.^45^ Bile acid metabolism is crucial in the regulation of lipid metabolism and the maintenance of cholesterol homeostasis (Figure 3).

### 3.1 Effect of Phytochemicals on Bile Acid Homeostasis

Studies have demonstrated that the regulatory effect of phytochemicals on enzymes involved in the hepatic synthesis and colonic metabolism of bile acids can be used to prevent the conditions caused by excess cholesterol. The application of phytochemicals to promote bile acid synthesis, metabolism, and excretion and reduce hepatic cholesterol levels has thus been suggested as a potential strategy. Additionally, preventing the reabsorption of bile acids from the ileum should be considered.

Saikosaponins, oleanane derivatives found in *Radix Bupleuri*, exhibited a modulatory effect on cholesterol clearance in a recent study; cholesterol efflux was promoted by significant upregulation of the genes related to cholesterol transportation, such as *Abca6*, *Apof*, *Npc1*, *Pdzk1*, *Ttc39b*, and *Scarb1*. Furthermore, the rate-limiting enzymes responsible for bile acid biosynthesis and cholesterol sulfation, including *cyp7a1*, *hsd3b7*, and *sult2b1*, were upregulated by saikosaponin intervention.^46^ These findings indicate the ameliorative effect of saikosaponins on hepatic steatosis, partly via promotion of cholesterol clearance through cholesterol efflux and bile acid synthesis. Geniposide, mentioned earlier as promoting cholesterol metabolism, was also shown to induce bile acid synthesis and excretion. Both the protein and mRNA levels of CYP7a1, CYP27a1, CYP7b1, and CYP8b1 were upregulated in response to geniposide treatment, leading to hepatic bile acid synthesis via the consumption of hepatic cholesterol.^47^

The farnesoid X receptor (FXR) is the most important nuclear receptor for maintaining bile acid homeostasis, playing a specific role in suppressing bile acid synthesis and promoting its enterohepatic circulation.^48^ FXR can inhibit the synthesis of bile acid via its negative feedback regulation, but geniposide has been found to inhibit the hepatic FXR level, accompanied by its downstream targets, bZip Maf transcription factor (MAFG) and tyrosine phosphatase (SHP). As further evidence, increased levels of bile acids in urine and feces suggest that bile acid excretion is promoted by geniposide intervention. In addition, the reduction in ileum FXR, ileal bile acid-binding protein (I-BABP), and apical sodium-dependent bile acid transporter (ASBT) suggests that geniposide intervention reduces bile acid reabsorption as a form of protection against these acids.^47^

The studies mentioned in this section suggest that the maintenance of bile acid levels is an effective strategy to prevent cholesterol-related diseases. Promoted bile acid synthesis should be accompanied by its increased excretion and/or the reduced resorption of colonic bile acid to prevent bile acid accumulation in the liver and simultaneously clear the level of hepatic cholesterol.

### 3.2 Effect of Phytochemicals on Bile Acid Recomposition and Recovery/Reabsorption

Bile acid is known to facilitate the digestion and absorption of lipids, and more importantly, regulate cholesterol homeostasis.^49^ Therefore, blindly promoting bile acid excretion could have a negative effect on the host. The strategic recomposition of the bile acid pool and promotion of bile acid reabsorption can, thus, maintain the host’s health.

Although the above-mentioned study reveals geniposide’s inhibitory effect on hepatic FXR in mice fed a high-fat diet, its promoting effect on FXR has been observed in bile duct ligation-induced cholestasis in mice models. Research has shown that the binding of geniposide to sirtuin 1 (SIRT1) increases the deacetylation of hepatic FXR and restores bile acid profiles/contents, especially those of chenodeoxycholic acid (CDCA), tauroursodeoxycholic acid (TUDCA), and taurochenodeoxycholic acid (TCDCA), ultimately ameliorating liver fibrosis.^50^ Therefore, phytochemicals might exhibit distinct effects under different disease conditions, and their benefits should be further clarified. Li et al. (2023) have reported similar results, demonstrating that vine tea extract restores hepatic and ileum FXR and ASBT—inhibiting bile acid synthesis and promoting its transport and reabsorption—and can serve as a means to improve hepatic injury.^51^ Scholars have also reported Forsythiaside A, the main active compound of *Forsythia suspensa* (Thunb.) Vahl, has a similar effect on hepatic bile acid synthesis in a CCl_4_-induced liver fibrosis mouse model. Forsythiaside A significantly reversed the suppression of the mRNA related to hepatic FXR-mediated bile acid regulation—CYP7A1, SHP, and liver receptor homologue-1 (LRH-1)—and transport–bile salt export pump (BSEP), multidrug resistance protein 4 (MRP4), Na^+^-taurocholate cotransporting polypeptide (NTCP), and organic anion transporting polypeptide-1 (OATP-1)—to restore the normal levels.^52^ Forsythiaside A intervention also reversed all CCl_4_-induced abnormal changes in bile acid levels.

The major characteristic of cholestasis is the accumulation of hepatic bile, and the activation of FXR to impede bile acid synthesis and promote its transportation is considered to be a promising strategy for maintaining bile acid homeostasis. Researchers have reported that *Dolomiaea souliei* (Franch.) C. Shih, a herbal medicine with costunolide as a major constituent, structurally interacts with FXR to prevent cholestasis.^53^ The binding of the compound to FXR significantly activated the FXR/SHP pathway; inhibited CYP7A1 and CYP27A1 to control the bile acid synthetic process; and upregulated BSEP, MRP2, and NTCP to promote bile acid transport, consequently ameliorating bile accumulation in the liver.

G protein-coupled bile acid receptor 1 (TGR5) is a transmembrane G-protein-coupled receptor (GPCR) for bile acids. Other than FXR, TGR5 has also been identified as a regulatory target of bile acid and may facilitate the occurrence of lipolysis and energy consumption.^54^ Research has indicated that red ginseng extract promotes ASBT membrane localization via TGR5 activation, resulting in bile acid uptake in mice intestines.^55^ The effect of red ginseng supplementation was reflected in the serum, white adipose tissue, and ileum bile acid profiles, although no significant changes in the composition of hepatic bile acid were observed. In another study, ginsenoside compound K activated TGR5 in the L-cells of the intestinal epithelium, possibly via the gut microbiota–bile acid axis.^56^ The intervention of compound K significantly facilitated the growth of *Akkermansia*, *Lactobacillaceae*, *Lachnospiraceae*, and *Ruminococcaceae* and simultaneously reduced the abundance of *Bacteroidaceae* and *Enterococcaceae*. Moreover, the deoxycholic acid (DCA) and lithocholic acid (LCA) levels increased significantly after treatment with compound K, which subsequently activated TGR5. In addition, the upregulation of CYP7B1 and CYP27A1 and downregulation of CYP8B1 occurred due to changes in the bile acid composition.

Both the activation of FXR and TGR5 and the inhibition of FXR by phytochemicals promote the maintenance of bile acid homeostasis. These results indicate that removing their expression might lead to negative effects. Therefore, phytochemicals and their regulatory effects are preferred over drugs in preventing side effects.

## 4 Relation of Cholesterol Regulation to Bile Acids via Gut Microbial Modulation

Many reports have indicated the correlation between the microbiome and the incidence of cardiovascular disease.^57,58^ Research has shown that the depletion of gut microbiota increases intestinal cholesterol uptake and cholesterol biosynthesis in the liver.^59^ In another study, the microbial conversion of cholesterol into coprostanol beneficially reduced intestinal and serum cholesterol levels, revealing the crucial role of gut microbiota.^60^ It is also understood that around 95% of bile acids are reabsorbed and recycled via enterohepatic circulation, while the remaining amount enters the colon and is dehydroxylated into DCA and LCA by gut microbes.^61^ FXR is activated by ligands, especially hydrophobic bile acids such as CDCA, followed by LCA and DCA (to a similar extent) and cholic acid (CA; with a lower potency).^62^ The activation of FXR is negatively correlated to CYP7A1, CYP8B1, CYP27A1, and CYP7B1, which are involved in inducing bile acid synthesis.^62^ Therefore, the colonic bile acid composition significantly influences the rate of hepatic bile acid synthesis.

### 4.1 Effect of Phytochemicals on Cholesterol/Bile Acid-Related Genes through Gut Microbiota

Moringa-Fu brick tea is produced via cofermentation technology combining the dark tea and moringa leaves, and its health benefits in regard to metabolic disorders, arising from its polyphenol and theabrownin content, have been previously reported.^63^ Recent studies have also indicated its effectiveness in reducing hepatic cholesterol via the regulation of bile acid metabolism (including CYP7A1 and CYP27A1 via the reduction of intestinal FXR); notably, the abundance of gut microbes associated with bile salt hydrolase (BSH) activity was regulated by Moringa-Fu brick tea extract.^63^ Some gut microbes, including *Akkermansia*, *Clostridium*, *Bacteroides*, *Roseburia*, *Parabacteroides*, and *Prevotella*, are positively correlated with serum high-density lipoprotein-cholesterol (HDL-C) and hepatic expressions of CYP7A1 and CYP27A1. The effect of chin brick tea, *Camellia sinensis* (L.) Kuntze, on modulating intestinal flora and, consequently, altering the composition of bile acid has also been recently reported. Polyphenols in the aqueous extract of chin brick tea (rich in catechin-like compounds) significantly increased the primary/secondary bile acid ratio. Specifically, *Bacteroides* positively correlated to nonalcoholic fatty liver disease (NAFLD) were positively correlated with DCA and ω-muricholic acid (ωMCA), while *Lactobacillus* exhibited a negative correlation with these, serum LDL-C/HDL-C, and the hepatic total cholesterol (TC).^64^ These studies suggest the modulatory effect of teas on gut microbial composition, which can consequently affect bile acid synthesis and metabolism. Therefore, further investigations are required to clarify the correlation between the composition of the bile acid and the abundance of specific gut microbes.

In addition to tea, other plant-based supplements display regulatory effects on the gut microbiota. Flaxseed powder, a commercial source of dietary α-linolenic acid, exhibits an ameliorative effect on high-fat-diet (HFD)-induced NAFLD, possibly by regulating gut microbiota and bile acid metabolism. Increases in the relative abundances of *Parasutterella*, *Lachnospiraceae_NK4A136_group*, and *[Eubacterium]_xylanophilum_group* have been reported in flaxseed-supplemented groups, accompanied by repression in the relative abundances of *Coriobacteriaceae_UCG-002*, *Erysipelatoclostridium*, and *[Eubacterium]_fissicatena_group*. A positive correlation has been reported between some bile acid metabolites and *Erysipelatoclostridium* and *[Eubacterium]_fissicatena_group*.^65^ Notably, changes in intestinal bile acid have led to the activation of FXR and resulted in a decrease in hepatic *cyp7a1* and *cyp8b1*, which control bile acid synthesis.

Disruptions to bile acid synthesis and metabolism may contribute to liver disease due to HFD intake. In an ovariectomized-induced dyslipidemia rat model, *Radix Angelica dahuricae* extract was found to interact with the proteins related to bile acid signaling pathways, including FXR, LXR, and PPARα. *Radix Angelica dahuricae* is a Chinese traditional medicine with furanocoumarins as active compounds, including imperatorin, isoimperatorin, oxypeucedanin hydrate, and oxypeucedanin;^66^ its extract increased BSH levels in the liver and cecum and resulted in the recomposition of bile acid. Significantly elevated levels of *Lactobacillus reuteri*, *Ruminococcus bromii*, and *Parabacteroides distasonis* were also identified in the group treated with *Radix Angelica dahuricae* extract.^66^ Generally, the bile acid pool is composed of both primary bile acids, including CA and CDCA, and secondary bile acids, such as DCA and LCA. The abundance of *Lactobacillus reuteri* was positively correlated to CA levels but negatively correlated to taurohyodeoxycholic acid (THDCA). In addition, *Ruminococcus bromii* showed a negative relationship with taurocholic acid (TCA), TUDCA, and THDCA but promoted the level of primary bile acids (CDCA) in humans.

Another study demonstrated the modulatory effect of a traditional Chinese formula comprising several traditional herbs on gut microbiota and revealed the correlation between some gut microbes and bile acids. *Akkermansia*, *Allobaculum*, *Bilophila*, *Clostridium*, and *Lactobacillus* were positively correlated to hepatic unconjugated bile acids and negatively correlated to conjugated ones; these gut microbes were positively correlated to the primary bile acids in feces and negatively correlated to secondary ones. Notably, *Coprococcus* and *Halomonas* showed opposite correlations with *Akkermansia*, *Allobaculum*, *Bilophila*, *Clostridium*, and *Lactobacillus*.^67^

### 4.2 Effect of Phytochemicals on Bile Acid Profiles through Gut Microbes

Berberine, an alkaloid isolated from the rhizome of *Coptis chinensis*, has recently been reported to improve gut microbiota depletion in a mouse colitis model by facilitating the growth of the *Lactobacillus* and *Roseburia* genera; this resulted in an increase in the colonic levels of CDCA and DCA, which activated colonic FXR and inhibited hepatic CYP7A1, leading to the control of bile acid synthesis. Reports have indicated that FXR/fibroblast growth factor 15 (FGF15) activation occurs via the intervention of *Tripterygium hypoglaucum* (Levl.) Hutch extract, a source of diterpenoids; the activation leads to a reduction in free bile acids and an increase in conjugated bile acids.^68^ The changes in the bile acid composition are listed in Table 2.

**Table 2:**
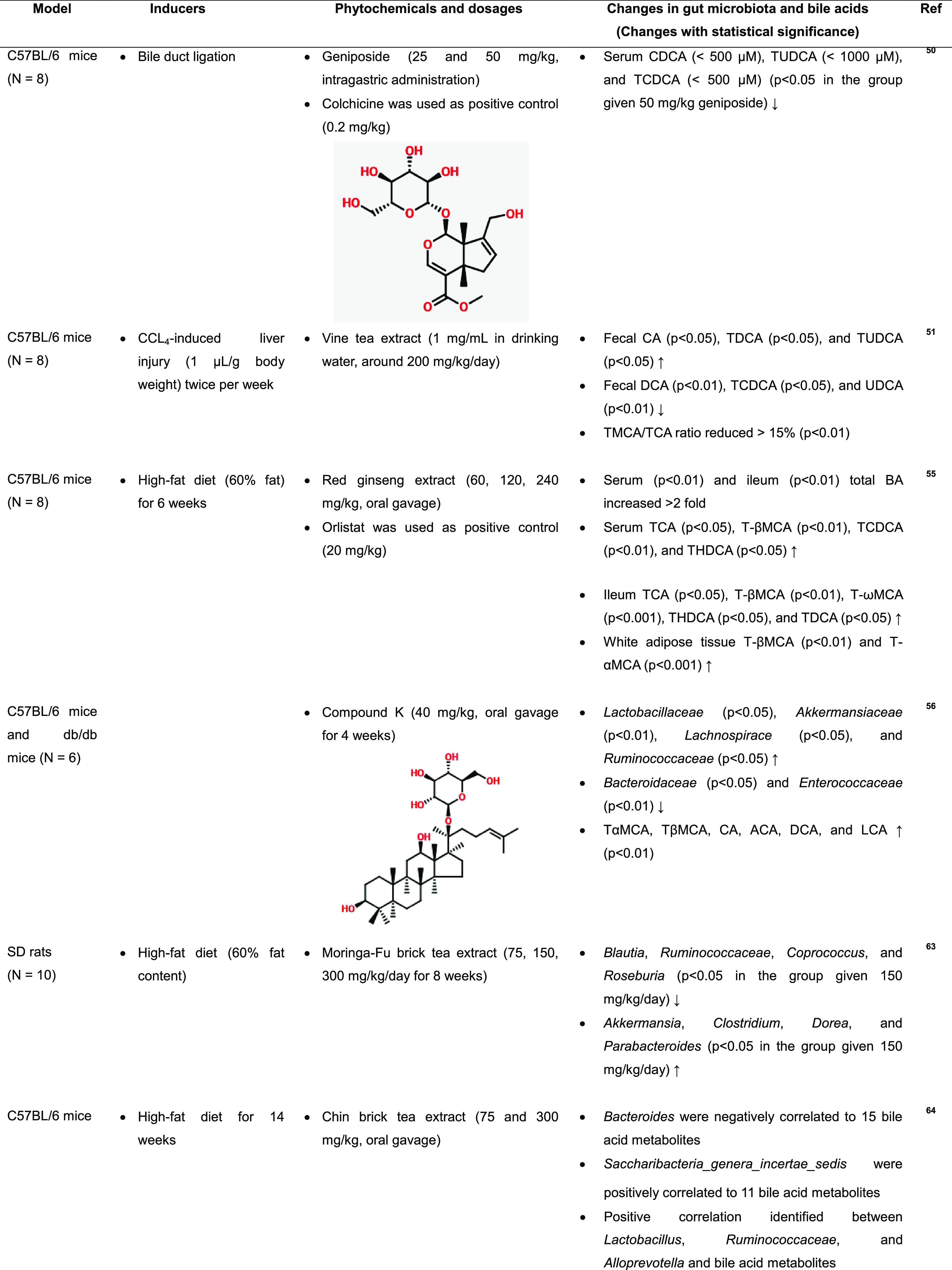

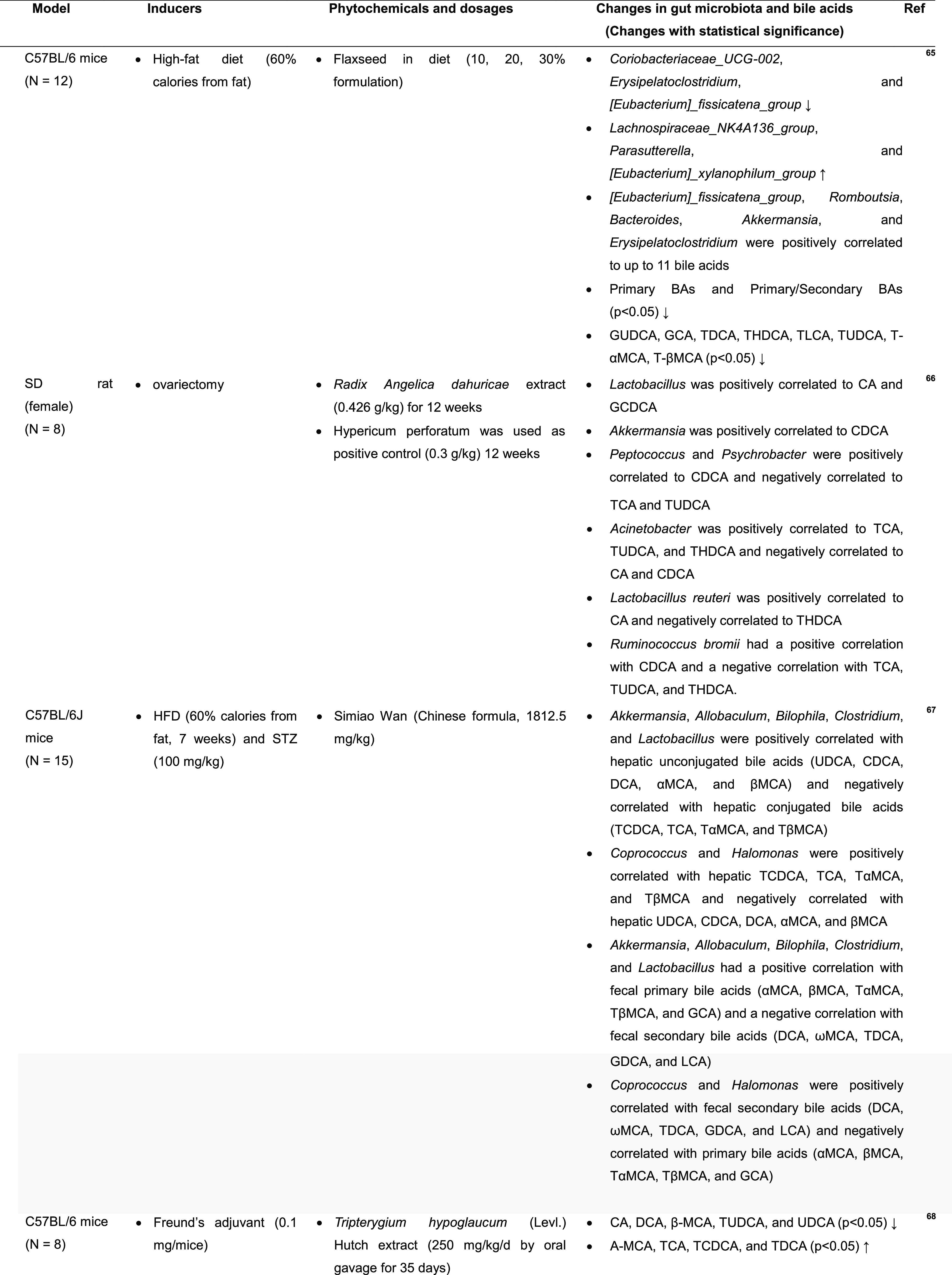

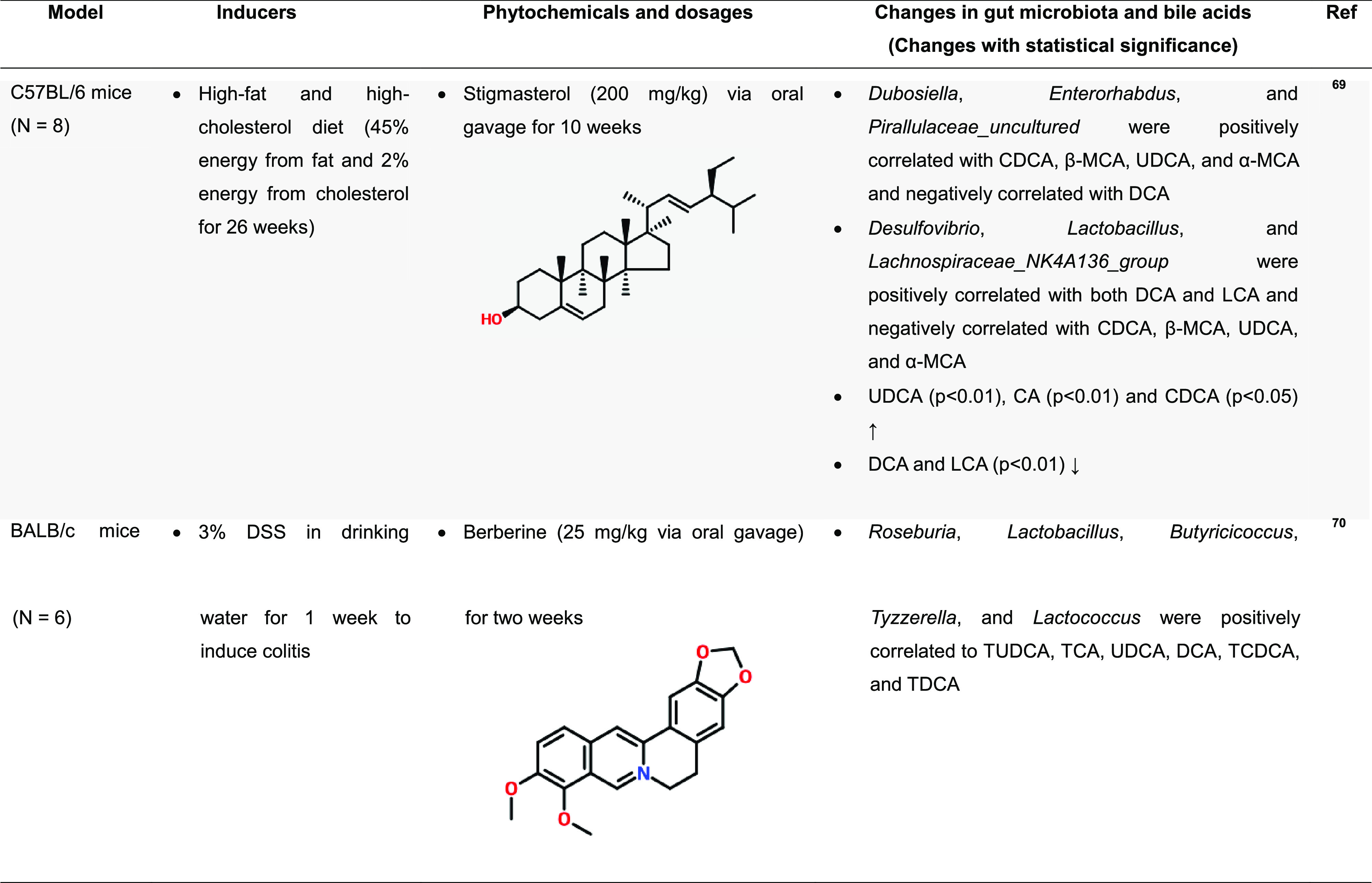
Effect of Phytochemicals on Bile Acid and Gut Microbial Composition

#### 4.2.1 Recomposition of Gut Microbial Profiles Promoting Bile Acid Synthesis

The above-mentioned studies demonstrate the suppression of bile acid synthesis to prevent metabolic diseases and avoid damage by bile acids. In contrast, other studies have suggested the enhancement of bile acid synthesis as a strategy to reduce hepatic cholesterol levels. The phytosterol stigmasterol, a natural steroid, has been widely studied and exhibits antihyperlipidemic and cholesterol-lowering properties. Stigmasterol intervention has not only reduced hepatic free cholesterol and 25-hydroxycholesterol levels but has also promoted alternative bile acid synthetic pathways (*cyp27a1*, *cyp7b1*, and *bsep* levels) and, subsequently, enhanced the fecal excretion of bile acids.^69^ Specifically, the colonic levels of hydrophilic bile acids (ursodeoxycholic acid [UDCA], CA, α-MCA, β-MCA, and CDCA) were significantly increased in the stigmasterol intervention group, while hydrophobic bile acids, such as DCA and LCA, were notably reduced, facilitating the excretion of bile acids via feces. The alterations in bile acids could be due to the reshaping of the gut microbiota by stigmasterol, which can significantly increase the Chao1 index and modulate the gut microbial compositions. The correlations between the different bile acids and gut microbes reviewed in this study are presented in Table 2.

## 5 Summary

Cholesterol is pivotal in the biological functioning of the body, but the Westernization of dietary patterns has promoted the incidence of metabolic diseases related to cholesterol dysregulation. Although a plethora of studies have focused on the potential effects of dietary compounds on cholesterol-related diseases, the outcomes are subtle. Studies on the effect of traditional Chinese medicines have led to some striking results, although there is a lack of sufficient scientific evidence to confirm and substantiate their significance in terms of disease improvement. Therefore, further studies should be conducted to identify the primary components responsible for the effects attributed to traditional Chinese medicines. Additionally, it is crucial to investigate whether these effects result from a synergistic interaction among the multiple beneficial components. More importantly, the specific underlying mechanisms that could be either promoted or inhibited by these components need to be further elucidated. Nevertheless, these phytochemicals could still serve as promising candidates to overcome the shortcomings of already-developed drugs (i.e., their side effects). Cholesterol remains important in the functioning of the body and thus should not be completely eliminated from the body by drugs; therefore, retuning and improving cholesterol levels could represent better strategies, though these present challenges, to some extent, for developed drugs. Furthermore, complications arising from gut microbiota, which, to a large extent, are modulated but not manipulated, because gut microbiota are vulnerable to change. Thus, perfectly improving cholesterol-related diseases with drugs is virtually impossible. In summary, studies on dietary compounds for cholesterol regulation cannot be neglected, and the development of therapeutic adjuvants for cholesterol dysregulation can be addressed in future studies.

## List of Abbreviations

*Acly*: ATP citrate lyase gene
AMPK: AMP-activated protein kinase
ASBT: apical sodium-dependent bile acid transporter
BAs: bile acids
BSEP: bile salt export pump
CA: cholic acid
CDCA: chenodeoxycholic acid
C/EBP: CCAAT/enhancer binding protein
DCA: deoxycholic acid
ECM1: extracellular matrix protein 1
FASN: fatty acid synthase
FDFT1: farnesyl-diphosphate farnesyltransferase 1
FGF15: fibroblast growth factor 15
FXR: farnesoid X receptor
GPAM: glycerol-3-phosphate acyltransferase
GPCR: G protein-coupled receptor
HDACs: histone deacetylases
HDL-C: high-density lipoprotein-cholesterol
HFD: high-fat diet
HMG-CoA: 3-hydroxy-3-methylglutaryl-CoA (produced from acetoacetyl-CoA)
HMGCR: 3-hydroxy-3-methylglutaryl-coenzyme A reductase
HMGCS1: 3-hydroxy-3-methylglutaryl-CoA synthase 1
HNF-1: hepatocyte nuclear factor-1
I-BABP: ileal bile acid-binding protein
LCA: lithocholic acid
LCAT: lecithin-cholesterol acyltransferase
LDL-C: low-density lipoprotein-cholesterol
LDLR: low-density lipoprotein receptor
LRH-1: liver receptor homologue-1
LSS: lanosterol synthase
LXR: liver X receptor
MAFG: bZip Maf transcription factor
MCA: muricholic acid
MRP4: multidrug resistance protein 4
MVD: mevalonate diphosphate decarboxylase
NAFLD: nonalcoholic fatty liver disease
NPC1L1: NPC1-like intracellular cholesterol transporter 1
NTCP: Na^+^-taurocholate cotransporting polypeptide
OATP: organic anion transporting polypeptide
ox-LDL: oxidized low-density lipoprotein
PCSK9: proprotein convertase subtilisin/kexin type 9
PPARs: peroxisome proliferator-activated receptors
RCT: reverse cholesterol transport
RUFY1: RUN and FYVE domain-containing protein 1
SCD: stearoyl-CoA desaturase
SHP: tyrosine phosphatase
SIRT1: sirtuin 1
SQLE: squalene epoxidase
SR-BI: scavenger receptor class B type I
SREBPs: sterol regulatory element-binding proteins
TC: total cholesterol
TCDCA: taurochenodeoxycholic acid
TGR5: G protein-coupled bile acid receptor 1
THDCA: taurohyodeoxycholic acid
TUDCA: tauroursodeoxycholic acid
UDCA: ursodeoxycholic acid

## References

[ref1] Ritchie S. A.; Connell J. M. C. The link between abdominal obesity, metabolic syndrome and cardiovascular disease. Nutr Metab Cardiovasc Dis 2007, 17, 319–326. 10.1016/j.numecd.2006.07.005. 17110092

[ref2] Huang P. L. A comprehensive definition for metabolic syndrome. Dis Model Mech 2009, 2, 231–237. 10.1242/dmm.001180. 19407331 PMC2675814

[ref3] Ezeh K. J.; Ezeudemba O. Hyperlipidemia: A review of the novel methods for the management of lipids. Cureus 2021, 13, e16412 10.7759/cureus.16412. PMC8364429 34401212

[ref4] Song Y.; Liu J.; Zhao K.; Gao L.; Zhao J. Cholesterol-induced toxicity: An integrated view of the role of cholesterol in multiple diseases. Cell Metab. 2021, 33, 1911–1925. 10.1016/j.cmet.2021.09.001. 34562355

[ref5] Bhatnagar D.; Soran H.; Durrington P. N. Hypercholesterolaemia and its management. BMJ. 2008, 337, a993 10.1136/bmj.a993. 18719012

[ref6] Ramkumar S.; Raghunath A.; Raghunath S. Statin therapy: Review of safety and potential side effects. Acta Cardiol. Sin. 2016, 32, 631–639. 10.6515/acs20160611a. 27899849 PMC5126440

[ref7] Islam S. U.; Ahmed M. B.; Ahsan H.; Lee Y.-S. Recent molecular mechanisms and beneficial effects of phytochemicals and plant-based whole foods in reducing LDL-C and preventing cardiovascular disease. Antioxidants (Basel) 2021, 10, 784 10.3390/antiox10050784. PMC8157003 34063371

[ref8] Dadkhah Tehrani S.; Shojaei M.; Bagherniya M.; Pirro M.; Sahebkar A. The effects of phytochemicals on serum triglycerides in subjects with hypertriglyceridemia: A systematic review of randomized controlled trials. Phytother Res. 2023, 37, 1640–1662. 10.1002/ptr.7763. 36756995

[ref9] Salvamani S.; Gunasekaran B.; Shukor M. Y.; Abu Bakar M. Z.; Ahmad S. A. Phytochemical investigation, hypocholesterolemic and anti-atherosclerotic effects of Amaranthus viridis leaf extract in hypercholesterolemia-induced rabbits. RSC Adv. 2016, 6, 32685–32696. 10.1039/C6RA04827G.

[ref10] Duan Y.; Gong K.; Xu S.; Zhang F.; Meng X.; Han J. Regulation of cholesterol homeostasis in health and diseases: from mechanisms to targeted therapeutics. Signal Transduct. Target Ther. 2022, 7, 265 10.1038/s41392-022-01125-5. 35918332 PMC9344793

[ref11] Chen Z.-Y.; Jiao R.; Ma K. Y. Cholesterol-lowering nutraceuticals and functional foods. J. Agric. Food Chem. 2008, 56, 8761–8773. 10.1021/jf801566r. 18778072

[ref12] Song T.; Wang P.; Li C.; Jia L.; Liang Q.; Cao Y.; Dong P.; Shi H.; Jiang M. Salidroside simultaneously reduces de novo lipogenesis and cholesterol biosynthesis to attenuate atherosclerosis in mice. Biomed. Pharmacother. 2021, 134, 111137 10.1016/j.biopha.2020.111137. 33341055

[ref13] Pan L.; Lu Y.; Dai S.; Tang X.; Xiong L.; Liu Z.; Gong Y. The role of cholesterol in modifying the lipid-lowering effects of Fuzhuan brick-tea in Caenorhabditis elegans via SBP-1/SREBP. Food Sci. Hum. Wellness. 2023, 12, 2297–2305. 10.1016/j.fshw.2023.03.033.

[ref14] Dong Y.; Yu C.; Ma N.; Xu X.; Wu Q.; Lu H.; Gong L.; Chen J.; Ren J. MicroRNA-379–5p regulates free cholesterol accumulation and relieves diet induced-liver damage in db/db mice via STAT1/HMGCS1 axis. Mol. Biomed. 2022, 3, 25 10.1186/s43556-022-00089-w. 35945406 PMC9363541

[ref15] Ma X.; Bai Y.; Liu K.; Han Y.; Zhang J.; Liu Y.; Hou X.; Hao E.; Hou Y.; Bai G. Ursolic acid inhibits the cholesterol biosynthesis and alleviates high fat diet-induced hypercholesterolemia via irreversible inhibition of HMGCS1 in vivo. Phytomedicine 2022, 103, 154233 10.1016/j.phymed.2022.154233. 35671633

[ref16] Zhang K.; Shen F.; Lei W.; Han Y.; Ma X.; Lu Y.; Hou Y.; Liu W.; Jiang M.; Zhang T.; Bai G. Ligustilide covalently binds to Cys129 of HMGCS1 to ameliorate dyslipidemia. Biomed. Pharmacother. 2023, 166, 115323 10.1016/j.biopha.2023.115323. 37579692

[ref17] Leng E.; Xiao Y.; Mo Z.; Li Y.; Zhang Y.; Deng X.; Zhou M.; Zhou C.; He Z.; He J.; Xiao L.; Li J.; Li W. Synergistic effect of phytochemicals on cholesterol metabolism and lipid accumulation in HepG2 cells. BMC Complement. Altern. Med. 2018, 18, 122 10.1186/s12906-018-2189-6. 29622007 PMC5887216

[ref18] Dong X.; Zhu Y.; Wang S.; Luo Y.; Lu S.; Nan F.; Sun G.; Sun X. Bavachinin inhibits cholesterol synthesis enzyme FDFT1 expression via AKT/mTOR/SREBP-2 pathway. Int. Immunopharmacol. 2020, 88, 106865 10.1016/j.intimp.2020.106865. 32827918

[ref19] Xiao M.-Y.; Li F.-F.; Xie P.; Qi Y.-S.; Xie J.-B.; Pei W.-J.; Luo H.-T.; Guo M.; Gu Y.-L.; Piao X.-L. Gypenosides suppress hepatocellular carcinoma cells by blocking cholesterol biosynthesis through inhibition of MVA pathway enzyme HMGCS1. Chem. Biol. Interact. 2023, 383, 110674 10.1016/j.cbi.2023.110674. 37604220

[ref20] Xu X. Y.; Choi H. S.; Park S. Y.; Kim J.-K.; Seo K. H.; Kim H.; Kim Y.-J. Hibiscus syriacus L. cultivated in callus culture exerts cytotoxicity in colorectal cancer via Notch signaling-mediated cholesterol biosynthesis suppression. Phytomedicine 2022, 95, 153870 10.1016/j.phymed.2021.153870. 34896899

[ref21] Wang J.; Chen Y.; Luo Z.; Huang Q.; Zhang Y.; Ning H.; Liu S.; Wang J.; Han X. Citri Reticulatae Pericarpium-Reynoutria japonica Houtt. herb pair suppresses breast cancer liver metastasis by targeting ECM1-mediated cholesterol biosynthesis pathway. Phytomedicine 2023, 116, 154896 10.1016/j.phymed.2023.154896. 37247588

[ref22] Guilbaud E.; Barouillet T.; Ilie M.; Borowczyk C.; Ivanov S.; Sarrazy V.; Vaillant N.; Ayrault M.; Castiglione A.; Rignol G.; Brest P.; Bazioti V.; Zaitsev K.; Lebrigand K.; Dussaud S.; Magnone V.; Bertolotto C.; Marchetti S.; Irondelle M.; Goldberg I.; Huby T.; Westerterp M.; Gautier E. L.; Mari B.; Barbry P.; Hofman P.; Yvan-Charvet L. Cholesterol efflux pathways hinder KRAS-driven lung tumor progenitor cell expansion. Cell Stem Cell 2023, 30, 800–817. 10.1016/j.stem.2023.05.005. 37267915

[ref23] Chen Y. Y.; Ren C. F.; Wen S. Y. Polyphyllin D induces G2/M cell cycle arrest via dysfunction of cholesterol biosynthesis in liver cancer cells. Biomed. Environ. Sci. 2023, 36, 94–98. 10.3967/bes2023.009. 36650685

[ref24] Mortensen M. B.; Dzaye O.; Bøtker H. E.; Jensen J. M.; Maeng M.; Bentzon J. F.; Kanstrup H.; Sørensen H. T.; Leipsic J.; Blankstein R.; Nasir K.; Blaha M. J.; Nørgaard B. L. Low-density lipoprotein cholesterol is predominantly associated with atherosclerotic cardiovascular disease events in patients with evidence of coronary atherosclerosis: The western Denmark heart registry. Circulation 2023, 147, 1053–1063. 10.1161/CIRCULATIONAHA.122.061010. 36621817 PMC10073288

[ref25] Qian Y.-W.; Schmidt R. J.; Zhang Y.; Chu S.; Lin A.; Wang H.; Wang X.; Beyer T. P.; Bensch W. R.; Li W.; Ehsani M. E.; Lu D.; Konrad R. J.; Eacho P. I.; Moller D. E.; Karathanasis S. K.; Cao G. Secreted PCSK9 downregulates low density lipoprotein receptor through receptor-mediated endocytosis. J. Lipid Res. 2007, 48, 1488–1498. 10.1194/jlr.M700071-JLR200. 17449864

[ref26] Ferrarese I.; Giovanna Lupo M.; Rossi I.; Sut S.; Loschi F.; Allegrini P.; Riva A.; Ferri N.; Dall’Acqua S. Bergamot (Citrus bergamia) peel extract as new hypocholesterolemic agent modulating PCSK9 expression. J. Funct. Foods 2023, 108, 105724 10.1016/j.jff.2023.105724.

[ref27] Dou X.; Zhou Z.; Ren R.; Xu M. Apigenin, flavonoid component isolated from Gentiana veitchiorum flower suppresses the oxidative stress through LDLR-LCAT signaling pathway. Biomed. Pharmacother. 2020, 128, 110298 10.1016/j.biopha.2020.110298. 32504920

[ref28] Li X.; Hu X.; Pan T.; Dong L.; Ding L.; Wang Z.; Song R.; Wang X.; Wang N.; Zhang Y.; Wang J.; Yang B. Kanglexin, a new anthraquinone compound, attenuates lipid accumulation by activating the AMPK/SREBP-2/PCSK9/LDLR signalling pathway. Biomed. Pharmacother. 2021, 133, 110802 10.1016/j.biopha.2020.110802. 33202286

[ref29] Siddiq A. A.; Martin A. Crocetin exerts hypocholesterolemic effect by inducing LDLR and inhibiting PCSK9 and Sortilin in HepG2 cells. Nutr. Res. (N.Y.) 2022, 98, 41–49. 10.1016/j.nutres.2021.08.005. 35093763

[ref30] Kim Y.-S.; Kim H.-R.; Antonisamy P.; Lee Y.-R.; Lee G.; Jung H.-J.; Kwon K.-B. Amomum villosum Lour. Fruit extract mitigates hyperlipidemia through SREBP-2/LDLR/HMGCR signaling in high-cholesterol diet-fed mice. J. King Saud Univ. Sci. 2022, 34, 102230 10.1016/j.jksus.2022.102230.

[ref31] Wang J.; Wang Y.-S.; Huang Y.-P.; Jiang C.-H.; Gao M.; Zheng X.; Yin Z.-Q.; Zhang J. Gypenoside LVI improves hepatic LDL uptake by decreasing PCSK9 and upregulating LDLR expression. Phytomedicine 2021, 91, 153688 10.1016/j.phymed.2021.153688. 34380071

[ref32] Yang Y.-N.; Wang Q.-C.; Xu W.; Yu J.; Zhang H.; Wu C. The berberine-enriched gut commensal Blautia producta ameliorates high-fat diet (HFD)-induced hyperlipidemia and stimulates liver LDLR expression. Biomed. Pharmacother. 2022, 155, 113749 10.1016/j.biopha.2022.113749. 36174380

[ref33] Ray A. G.; Choudhury K. R.; Chakraborty S.; Chakravarty D.; Chander V.; Jana B.; Siddiqui K. N.; Bandyopadhyay A. Novel mechanism of cholesterol transport by ABCA5 in macrophages and its role in dyslipidemia. J. Mol. Biol. 2020, 432, 4922–4941. 10.1016/j.jmb.2020.07.006. 32687853

[ref34] Sun Y.; Wang J.; Long T.; Qi X.; Donnelly L.; Elghobashi-Meinhardt N.; Esparza L.; Cohen J. C.; Xie X.-S.; Hobbs H. H.; Li X. Molecular basis of cholesterol efflux via ABCG subfamily transporters. Proc. Natl. Acad. Sci. U. S. A. 2021, 118, e2110483118 10.1073/pnas.2110483118. 34404721 PMC8403869

[ref35] Xie J.; Peng L.; wang T.; Yang C.; Chen N.; Feng X.; Wu T.; Xu T.; Chen Y. QiShenYiQi pill inhibits atherosclerosis by promoting reverse cholesterol transport PPARγ-LXRα/β-ABCA1 pathway. J. Ethnopharmacol. 2023, 315, 116684 10.1016/j.jep.2023.116684. 37230281

[ref36] Yan Y.-R.; Jia Z.-J.; Wang Y.; Xu F.-Q.; Zhou Q.-B. Huazhuotongmai decoction exerts anti-atherosclerotic effects by modulating the expression of ABCA1/SR-B1/PPAR-γ in vivo and in vitro. Phytomed. Plus. 2023, 3, 100436 10.1016/j.phyplu.2023.100436.

[ref37] He Y.; Tian W.; Zhang M.; Qiu H.; Li H.; Shi X.; Song S.; Wen C.; Chen J. Jieduquyuziyin prescription alleviates SLE complicated by atherosclerosis via promoting cholesterol efflux and suppressing TLR9/MyD88 activation. J. Ethnopharmacol. 2023, 309, 116283 10.1016/j.jep.2023.116283. 36898449

[ref38] Deng W.-Y.; Zhou C.-L.; Zeng M.-Y. Gypenoside XVII inhibits ox-LDL-induced macrophage inflammatory responses and promotes cholesterol efflux through activating the miR-182–5P/HDAC9 signaling pathway. J. Ethnopharmacol. 2024, 319, 117070 10.1016/j.jep.2023.117070. 37625608

[ref39] Wang G.; Gao J.-H.; He L.-H.; Yu X.-H.; Zhao Z.-W.; Zou J.; Wen F.-J.; Zhou L.; Wan X.-J.; Tang C.-K. Fargesin alleviates atherosclerosis by promoting reverse cholesterol transport and reducing inflammatory response. Biochim. Biophys. Acta, Mol. Cell Biol. Lipids 2020, 1865, 158633 10.1016/j.bbalip.2020.158633. 31988050

[ref40] Peng Y.; Xu J.; Zeng Y.; Chen L.; Xu X. L. Polydatin attenuates atherosclerosis in apolipoprotein E-deficient mice: Role of reverse cholesterol transport. Phytomedicine 2019, 62, 152935 10.1016/j.phymed.2019.152935. 31085374

[ref41] Yuan L.; Zhang F.; Jia S.; Xie J.; Shen M. Differences between phytosterols with different structures in regulating cholesterol synthesis, transport and metabolism in Caco-2 cells. J. Funct. Foods 2020, 65, 103715 10.1016/j.jff.2019.103715.

[ref42] Zhang R.; Han Y.; McClements D. J.; Xu D.; Chen S. Production, characterization, delivery, and cholesterol-lowering mechanism of phytosterols: A review. J. Agric. Food Chem. 2022, 70, 2483–2494. 10.1021/acs.jafc.1c07390. 35170307

[ref43] He W.-S.; Zhao L.; Yang H.; Rui J.; Li J.; Chen Z.-Y. Novel synthesis of phytosterol ferulate using acidic ionic liquids as a catalyst and its hypolipidemic activity. J. Agric. Food Chem. 2024, 72, 2309–2320. 10.1021/acs.jafc.3c09148. 38252882 PMC10835726

[ref44] He W.-S.; Zhu H.; Chen Z.-Y. Plant Sterols: Chemical and enzymatic structural modifications and effects on their cholesterol-lowering activity. J. Agric. Food Chem. 2018, 66, 3047–3062. 10.1021/acs.jafc.8b00059. 29521096

[ref45] Chiang J. Y. L.; Ferrell J. M.; Wu Y.; Boehme S. Bile acid and cholesterol metabolism in atherosclerotic cardiovascular disease and therapy. Cardiol Plus 2020, 5, 159–170. 10.4103/2470-7511.305419. 34350368 PMC8330388

[ref46] Zheng Q.; Li X.; Huang N.; Li F.; Ge J.; Wang D.; Sun R.; Liu R. Saikosaponins ameliorate hyperlipidemia in rats by enhancing hepatic lipid and cholesterol metabolism. J. Ethnopharmacol. 2023, 305, 116110 10.1016/j.jep.2022.116110. 36581162

[ref47] Liu J.; Li Y.; Sun C.; Liu S.; Yan Y.; Pan H.; Fan M.; Xue L.; Nie C.; Zhang H.; Qian H.; Ying H.; Wang L. Geniposide reduces cholesterol accumulation and increases its excretion by regulating the FXR-mediated liver-gut crosstalk of bile acids. Pharmacol. Res. 2020, 152, 104631 10.1016/j.phrs.2020.104631. 31911244

[ref48] Stofan M.; Guo G. L. Bile acids and FXR: Novel targets for liver diseases. Front. Med. 2020, 7, 544 10.3389/fmed.2020.00544. PMC7516013 33015098

[ref49] Staels B.; Fonseca V. A. Bile acids and metabolic regulation: mechanisms and clinical responses to bile acid sequestration. Diabetes Care 2009, 32 (Suppl 2), S237–S245. 10.2337/dc09-S355. 19875558 PMC2811459

[ref50] Qin T.; Hasnat M.; Wang Z.; Hassan H. M.; Zhou Y.; Yuan Z.; Zhang W. Geniposide alleviated bile acid-associated NLRP3 inflammasome activation by regulating SIRT1/FXR signaling in bile duct ligation-induced liver fibrosis. Phytomedicine 2023, 118, 154971 10.1016/j.phymed.2023.154971. 37494875

[ref51] Li Y.; Kong M.-W.; Jiang N.; Ye C.; Yao X.-W.; Zou X.-J.; Hu H.-M.; Liu H.-T. Vine tea extract ameliorated acute liver injury by inhibiting hepatic autophagy and reversing abnormal bile acid metabolism. Heliyon 2023, 9, e20145 10.1016/j.heliyon.2023.e20145. 37809393 PMC10559920

[ref52] Fu K.; Ma C.; Wang C.; Zhou H.; Gong L.; Zhang Y.; Li Y. Forsythiaside A alleviated carbon tetrachloride-induced liver fibrosis by modulating gut microbiota composition to increase short-chain fatty acids and restoring bile acids metabolism disorder. Biomed. Pharmacother. 2022, 151, 113185 10.1016/j.biopha.2022.113185. 35623173

[ref53] Meng F.; Zong W.; Wei X.; Tao Y.; Wang G.; Liao Z.; Chen M. Dolomiaea souliei ethyl acetate extract protected against α-naphthylisothiocyanate-induced acute intrahepatic cholestasis through regulation of farnesoid x receptor-mediated bile acid metabolism. Phytomedicine 2021, 87, 153588 10.1016/j.phymed.2021.153588. 34091148

[ref54] Duboc H.; Taché Y.; Hofmann A. F. The bile acid TGR5 membrane receptor: From basic research to clinical application. Dig Liver Dis 2014, 46, 302–312. 10.1016/j.dld.2013.10.021. 24411485 PMC5953190

[ref55] Li W.; Zhuang T.; Wang Z.; Wang X.; Liu L.; Luo Y.; Wang R.; Li L.; Huang W.; Wang Z.; Yang L.; Ding L. Red ginseng extracts ameliorate high-fat diet-induced obesity and insulin resistance by activating the intestinal TGR5-mediated bile acids signaling pathway. Phytomedicine 2023, 119, 154982 10.1016/j.phymed.2023.154982. 37531904

[ref56] Tian F.; Huang S.; Xu W.; Chen L.; Su J.; Ni H.; Feng X.; Chen J.; Wang X.; Huang Q. Compound K attenuates hyperglycemia by enhancing glucagon-like peptide-1 secretion through activating TGR5 via the remodeling of gut microbiota and bile acid metabolism. J. Ginseng Res. 2022, 46, 780–789. 10.1016/j.jgr.2022.03.006. 36312739 PMC9597441

[ref57] Al Samarraie A.; Pichette M.; Rousseau G. Role of the gut microbiome in the development of atherosclerotic cardiovascular disease. Int. J. Mol. Sci. 2023, 24, 5420 10.3390/ijms24065420. 36982492 PMC10051145

[ref58] Rahman M. M.; Islam F.; Or-Rashid M. H.; Mamun A. A.; Rahaman M. S.; Islam M. M.; Meem A. F. K.; Sutradhar P. R.; Mitra S.; Mimi A. A.; Emran T. B.; Fatimawali; Idroes R.; Tallei T. E.; Ahmed M.; Cavalu S. The gut microbiota (microbiome) in cardiovascular disease and its therapeutic regulation. Front Cell Infect Microbiol. 2022, 12, 903570 10.3389/fcimb.2022.903570. 35795187 PMC9251340

[ref59] Jia B.; Zou Y.; Han X.; Bae J.-W.; Jeon C. O. Gut microbiome-mediated mechanisms for reducing cholesterol levels: implications for ameliorating cardiovascular disease. Trends Microbiol. 2023, 31, 76–91. 10.1016/j.tim.2022.08.003. 36008191

[ref60] Bubeck A. M.; Urbain P.; Horn C.; Jung A. S.; Ferrari L.; Ruple H. K.; Podlesny D.; Zorn S.; Laupsa-Borge J.; Jensen C.; Lindseth I.; Lied G. A.; Dierkes J.; Mellgren G.; Bertz H.; Matysik S.; Krautbauer S.; Liebisch G.; Schoett H.-F.; Dankel S. N.; Fricke W. F. High-fat diet impact on intestinal cholesterol conversion by the microbiota and serum cholesterol levels. iScience 2023, 26, 107697 10.1016/j.isci.2023.107697. 37694136 PMC10485154

[ref61] Collins S. L.; Stine J. G.; Bisanz J. E.; Okafor C. D.; Patterson A. D. Bile acids and the gut microbiota: metabolic interactions and impacts on disease. Nat. Rev. Microbiol. 2023, 21, 236–247. 10.1038/s41579-022-00805-x. PMC12536349 36253479

[ref62] Ding L.; Yang L.; Wang Z.; Huang W. Bile acid nuclear receptor FXR and digestive system diseases. Acta Pharm. Sin B 2015, 5, 135–144. 10.1016/j.apsb.2015.01.004. PMC4629217 26579439

[ref63] Ou-yang J.; Li X.-p.; Liu C.-w.; Ou-yang J.; Tang J.-y.; Liu Q.; Huang J.-a.; Liu Z. Moringa-Fu brick tea extract attenuated high-fat diet-induced obesity via modulating bile acid metabolism and gut microbiota in rats. J. Funct. Foods 2023, 109, 105766 10.1016/j.jff.2023.105766.

[ref64] Jin C.; Zhou T.; Duan Z.; Deng Y.; Zhang X.; Xiao C.; He J.; He G.; Zhou Y.; Li S. Effect of chin brick tea [Camellia sinensis (L.) Kuntze] on lipid metabolism and inflammation by modulating intestinal flora and bile acids in mice with non-alcoholic fatty liver disease. J. Ethnopharmacol. 2024, 318, 116950 10.1016/j.jep.2023.116950. 37506781

[ref65] Yang C.; Yang L.; Yang Y.; Wan M.; Xu D.; Pan D.; Sun G. Effects of flaxseed powder in improving non-alcoholic fatty liver by regulating gut microbiota-bile acids metabolic pathway through FXR/TGR5 mediating. Biomed. Pharmacother. 2023, 163, 114864 10.1016/j.biopha.2023.114864. 37167728

[ref66] Chen L.; Liu Y.; Tang Z.; Song Z.; Cao F.; Shi X.; Xie P.; Wei P.; Li M. Radix Angelica dahuricae extract ameliorates oestrogen deficiency-induced dyslipidaemia in ovariectomized (OVX) rats by modulating the gut microbiota and bile acid signalling. Phytomedicine 2022, 107, 154440 10.1016/j.phymed.2022.154440. 36162241

[ref67] Chen Y.; Zhu L.; Hu W.; Wang Y.; Wen X.; Yang J. Simiao Wan modulates the gut microbiota and bile acid metabolism during improving type 2 diabetes mellitus in mice. Phytomedicine 2022, 104, 154264 10.1016/j.phymed.2022.154264. 35752076

[ref68] Zheng J.; Hu J.; Yang Y.; Xiong L.; Yang H.; Zhang Z.; Jiang N.; Liu H. Suppressive effect of Tripterygium hypoglaucum (Levl.) Hutch extract on rheumatoid arthritis in mice by modulating inflammasome and bile acid metabolism. Biomed. Pharmacother. 2023, 167, 115494 10.1016/j.biopha.2023.115494. 37734264

[ref69] Xin Y.; Li X.; Zhu X.; Lin X.; Luo M.; Xiao Y.; Ruan Y.; Guo H. Stigmasterol protects against steatohepatitis induced by high-fat and high-cholesterol diet in mice by enhancing the alternative bile acid synthesis pathway. J. Nutr. 2023, 153, 1903–1914. 10.1016/j.tjnut.2023.05.026. 37269906

[ref70] Sun X.; Zhang Y.; Cheng G.; Zhu T.; Zhang Z.; Xiong L.; Hu H.; Liu H. Berberine improves DSS-induced colitis in mice by modulating the fecal-bacteria-related bile acid metabolism. Biomed. Pharmacother. 2023, 167, 115430 10.1016/j.biopha.2023.115430. 37683590

[ref71] Sitaula S.; Burris T. P. Cholesterol and Other Steroids. In Encyclopedia of Cell Biology; Bradshaw R. A.; Stahl P. D., Eds.; Academic Press: Waltham, 2016; pp 173–179.

[ref72] Latimer J.; Batty J. A.; Neely R. D. G.; Kunadian V. PCSK9 inhibitors in the prevention of cardiovascular disease. J. Thromb Thrombolysis 2016, 42, 405–419. 10.1007/s11239-016-1364-1. 27095708 PMC5010583

[ref73] Salic K.; Kleemann R.; Wilkins-Port C.; McNulty J.; Verschuren L.; Palmer M. Apical sodium-dependent bile acid transporter inhibition with volixibat improves metabolic aspects and components of non-alcoholic steatohepatitis in Ldlr–/– Leiden mice. PLoS One 2019, 14, e0218459 10.1371/journal.pone.0218459. 31233523 PMC6590809

